# The Impact and Acceptance of Gamification by Learners in a Digital Literacy Course at the Undergraduate Level: Randomized Controlled Trial

**DOI:** 10.2196/52017

**Published:** 2024-08-23

**Authors:** Abeer Alnuaim

**Affiliations:** 1 Department of Computer Science and Engineering, College of Applied Studies and Community Service, King Saud University, P.O. Box 22459, Riyadh, 11495, Saudi Arabia, 966 114670000

**Keywords:** gamification, games, technology integration, information literacy, technology acceptance

## Abstract

**Background:**

In recent years, the integration of technology in education has revolutionized traditional learning paradigms. Digital literacy, a crucial skill in the 21st century, has become a vital aspect of modern education, enabling students to navigate, critically assess, and effectively use digital tools. As educators strive to boost engagement and learning outcomes, gamification has appeared as an auspicious pedagogical approach. By applying game mechanics to nongame contexts, gamification seeks to create a more immersive and digital learning experience.

**Objective:**

This research paper aims to investigate the impact and acceptance of gamification by learners in a digital literacy course at the undergraduate level.

**Methods:**

In a pre-post intervention study, 168 undergraduate students were randomly assigned either to the experimental group (gamification based) or control group (conventional) learning condition. Both groups of participants learned the same topics in digital literacy.

**Results:**

Empirical findings showed that participants from the experimental group had better academic performance in digital literacy than those who were not exposed to the game-based learning environment. The participants’ prior experience with gamification was not found to be a significant predictor of their acceptance of gamification in a digital literacy course.

**Conclusions:**

The study provides evidence supporting the potential benefits of gamification in enhancing digital literacy education and opens the door for further exploration and implementation of gamified learning approaches in higher education settings.

## Introduction

### Background

In today’s interconnected world, the demand for digital literacy has intensified across academic, professional, and personal domains. Digital literacy is a set of skills essential for 21st century individuals to use digital tools to support the achievement of goals in their life situations [1,2]. It has become not only a key factor in enabling participation in education and employment but also a means of interacting with the world. It encompasses the ability to access, analyze, evaluate, and create information using various digital applications. In the context of undergraduate education, cultivating digital literacy is essential for students to excel in their studies, conduct research, and adapt to the demands of the workforce in the information era. As universities strive to equip their students with these vital skills, innovative teaching approaches that enhance engagement and knowledge retention are warranted [3].

At the same time, incorporating gamification into pedagogical approaches has gained considerable attention [4,5]. The term gamification first appeared in 2008 and received growing significance since the 2010s [6]. In general, gamification refers to a process of augmenting services with (motivational) affordances to raise gameful experiences and promote behavioral outcomes [7]. In contrast to games, gamification is described by its serious purpose. It involves applying game design elements and mechanics in nongame contexts to enhance user engagement, motivation, and learning outcomes. According to Trinidad et al [8], gamification has swiftly appeared as one of the preferred persuasive technologies widely used with the aim of stimulating a positive change in the user’s behavior through game-like elements in nongame contexts. Likewise, Krath et al [6] argued that gamification is a great way to demonstrate goals and their germaneness, push users through directed tracks, provide users instant feedback, strengthen good performance, and streamline content to manageable tasks. It leverages the intrinsic motivational elements found in games to create a positive and engaging learning environment. Hamari et al [7] suggest that gamification has beneficial impacts; however, these effects greatly rely on the context in which it is used as well as on the individuals who practice it. Similar to this, Huang et al [9] argue that it can be difficult for educational academics and practitioners to decide when and how to apply gamification design elements.

Given the significant traction gained by gamification in recent years, several state-of-the-art gamified solutions and approaches have been developed and tested [10]. One widely used example of gamification for education is Kahoot. Kahoot! is a game-based learning platform that allows educators to create and deliver quizzes in a game format [11]. It incorporates leaderboards, points, and real-time feedback to create a competitive and engaging learning environment. Research indicates that Kahoot! enhances student participation and knowledge retention [12]. Similarly, Duolingo is a language-learning platform that uses gamification to teach foreign languages. It uses elements such as skill trees, streaks, and in-game currency to motivate learners. Studies have found that Duolingo is effective in improving language proficiency and maintaining learner interest [13].

Gamification is significantly correlated with game-based learning. Game-based learning is defined as the achievement of distinct learning objectives through game content and play and augmenting learning by including problem-solving spaces and challenges that offer learners, who are also players, with a feeling of achievement [6]. By incorporating game design elements such as points, badges, levels, leaderboards, and immediate feedback, gamified learning experiences can stimulate students’ curiosity and foster a sense of accomplishment [5,14]. Through gamification, educators aim to increase student motivation, participation, and knowledge retention by transforming learning from a passive experience into an active and enjoyable process [15,16]. Hamari and Homner have also recognized it as a promising method for instructional contexts for its motivational power [7]. The application of gamification has been successful in various educational contexts [9], including language learning [17,18], mathematics [19,20], and computer programming [21]. Its use in nongame contexts is associated with impacts on motivation, behavior (eg, academic achievement and engagement), and cognitive learning [6]. All of this demonstrates the potential of gamification to augment a digital literacy learning experience as well.

Even though recent research has made significant strides in this area, additional information about the integration of game aspects into educational materials is still required. Particularly, there is a dearth of coherent understanding on its use in the subject area of digital literacy. Particularly, there is a need to explore how adult learners perceive and accept gamification in the context of a digital literacy course at the undergraduate level in Saudi Arabia. By investigating the impact and acceptance of gamification in a digital literacy course at the undergraduate level, this research aims to shed light on its efficacy and potential for cultivating essential digital literacy. Addressing this gap in knowledge will provide valuable insights into the factors that influence Saudi students’ acceptance of gamification and the impact it has on their learning experience, thereby enabling educators and course designers to make informed decisions regarding the effective implementation of gamification strategies.

In the next section, we discuss the theoretical foundations for this study that helped us formulate our research questions and hypotheses.

### Theoretical Framework

In recent years, scientific papers have progressively investigated the use of different theoretical foundations such as motivation, behavior, and learning theories to explain the effects of certain gamification elements [6]. The theoretical footings of this study align with 2 pronounced frameworks in the field of education and technology: the self-determination theory (SDT) and the technology acceptance model (TAM). These theoretical foundations provide a comprehensive lens through which to understand the impact and acceptance of gamification in the context of digital literacy education at the undergraduate level. A brief overview of these theories and justification how they are aligned with this study is provided in the following sections.

#### Self-Determination Theory

Developed by Deci and Ryan, the SDT posits that individuals have innate psychological needs for autonomy, competence, and relatedness, which serve as essential motivators for behavior and engagement [22]. In the context of education, SDT suggests that learners are more likely to be intrinsically motivated and experience greater satisfaction and well-being when their psychological needs for autonomy, competence, and relatedness are supported [22].

The integration of gamification in educational settings can be viewed through the lens of the SDT, as it has the potential to fulfill learners’ psychological needs helping them experience intrinsic motivation, which drives them to engage in activities for the sheer enjoyment and interest in the task itself [22]. By providing learners with autonomy through choice and control over their learning paths, gamified experiences empower students to take ownership of their learning. Further, the challenge-based nature of game-based experiences foster feelings of competence as learners strive to achieve goals and overcome obstacles within the game environment. In addition, the social elements inherent in many gamified platforms facilitate a sense of relatedness by promoting collaboration, competition, and community among learners [6].

SDT is one of the well-known theories in the context of gamification. In the context of this study, the impact of gamification on learning outcomes can be better understood in terms of its alignment with the principles of SDT. Accordingly, by leveraging game mechanics to enhance autonomy, competence, and relatedness, gamification has the potential to promote intrinsic motivation and engagement [7] among undergraduate students in a digital literacy course.

#### Technology Acceptance Model

A popular framework for evaluating people’s attitudes and behavioral intentions toward embracing new technologies is Davis’s TAM [23]. According to TAM, perceived usefulness and perceived ease of use are key determinants of any individual’s intention to use a technology, which eventually influences his or her actual usage behavior. Perceived usefulness refers to the extent to which a person believes that using a particular technology will enhance their performance or productivity, while perceived ease of use pertains to the degree of effort required to use the technology effectively [24].

TAM has been extensively used in the context of educational technology to examine learners’ attitudes and behaviors toward various digital tools and platforms. Researchers can learn more about the elements influencing students’ acceptance and engagement with these technologies by evaluating how useful and simple they believe gamified learning environments to be [24,25]. In this study, TAM provides a theoretical framework for understanding learners’ acceptance of gamification in a digital literacy course. By investigating the perceived usefulness and ease of use of gamified learning experiences, the study seeks to elucidate the factors that contribute to students’ willingness to engage with and embrace gamification as a pedagogical approach.

By integrating the SDT and TAM frameworks into the research design and analysis, this study aims to provide a comprehensive understanding of the impact and acceptance of gamification by learners in a digital literacy course at the undergraduate level. In aligning our research design with the constructs of SDT, we focused on measuring students’ intrinsic motivation and perceived autonomy in the learning process. Similarly, the TAM informed our analysis of students’ acceptance of gamified learning tools, focusing on perceived ease of use and usefulness as key determinants. Furthermore, in interpreting our findings, we draw on both frameworks to discuss theoretical and practical implications of the study.

### Research Gap and Rationale

The growing significance of digital literacy in the modern world necessitates effective and engaging teaching strategies. Gamification has arisen as an encouraging approach that leverages the power of games to enhance learning experiences. While gamification shows promise as an innovative pedagogical approach, its impact and acceptance specifically in the context of digital literacy courses at the undergraduate level remain relatively unexplored. Existing research primarily focuses on the effects of gamification in K-12 education or specialized domains [6,26,27]. For example, Dehghanzadeh et al [25] concentrated on gamification-supported learning in K-12 settings, and Tan et al [27] described several mathematics gamification instances to enrich algebra teaching at school levels. Thus, there is a need to delve into the unique challenges and opportunities of implementing gamification in higher education, particularly in digital literacy courses. By investigating the impact of gamification on learning outcomes, the study can offer insights into the effectiveness of this approach in improving digital literacy among undergraduate students. Moreover, understanding students’ perceptions and acceptance of gamification can provide crucial feedback for educators and instructional designers to refine gamified learning experiences in digital literacy courses. The study’s outcomes may also inform policy decisions regarding the integration of gamification and technology in higher education curricula, paving the way for more engaging and effective teaching practices in the digital age.

### Research Questions

This study seeks to contribute to the existing body of knowledge by examining the following research questions:

1. What is the impact of gamification on the learning outcomes of students in a digital literacy course at the undergraduate level?
2. What factors affect the acceptance of learners toward gamified learning experiences in a digital literacy course?
3. How does learners’ prior experience with gamified learning environments affect their acceptance of gamification in a digital literacy course at the undergraduate level?

Based on the above research questions, the following hypotheses are proposed:

- H1: Gamification has a significant positive effect on learning outcomes of students in a digital literacy course at the undergraduate level.
- H2: Age and the major of students’ degree programs are significant factors influencing learners’ acceptance of gamification in a digital literacy class.
- H3: Learners’ prior experience with gamified learning environments has a significant effect on their acceptance of gamification in a digital literacy course.

## Methods

### Participants

Using convenience sampling, this study involved a diverse group of 168 undergraduate students enrolled in a digital literacy course at a Saudi university. While convenience sampling may not provide a representative sample of the population, it is the most practical in small-scale studies or when studying hard-to-reach populations [28]. Likewise, this study justifies this approach by emphasizing the need to assess the initial impact of a specific pedagogical approach in a real-world setting before considering broader applications or generalizations. All of these participants were native Arabic speakers. The students’ age ranged from 18 to 22 years with an average of 20.6 years.

Intervention students participated in the 16-week digital literacy course. The course is offered to the students enrolled in various undergraduate programs. The participants were distributed into 2 groups for a true experimental design [29,30]. There were 84 students in the experimental group and 84 in the control group. Participants in this study were randomly assigned to either the control group or the experimental group using a computerized randomization procedure.

The process of randomization of the participants into the 2 groups started with the creation of a list of all participating students, including their names and class roll numbers. A random number generator was used to create a sequence of random numbers corresponding to the total number of participants (N=168). This sequence was generated using a computer software program designed for randomization to eliminate any potential bias. The first 84 numbers in the sequence were assigned to the experimental group, and the remaining 84 numbers were assigned to the control group. The allocation process was conducted by a researcher who was not involved in the instructional process to ensure blinding. This helped minimize any potential bias in group assignment. This approach ensured that each participant had an equal chance of being placed in either group, thereby minimizing potential biases and confounding variables. By implementing random assignment, the study aimed to create comparable groups, ensuring that any observed differences in digital literacy skills and engagement could be attributed to the gamified intervention rather than to pre-existing differences between the participants.

### Experiment Process

**Figure 1.**
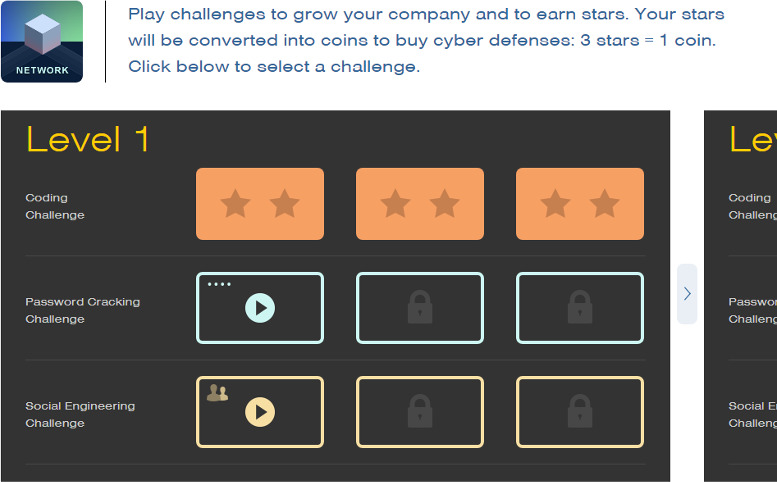
Snapshot 1 of “cybersecurity lab,” a cybersecurity game.

**Figure 2.**
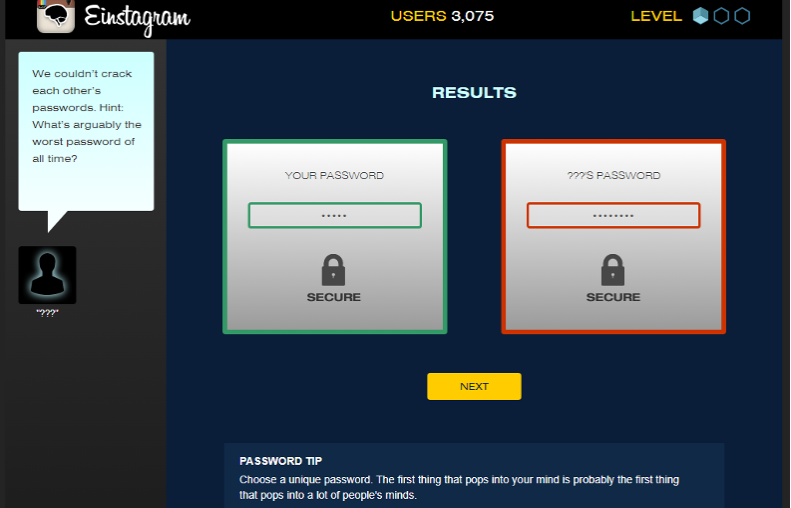
Snapshot 2 of “cybersecurity lab,” a cybersecurity game.

The teaching process took place partially in a lecture room and a computer lab. The duration of each lesson was 1 hour. The whole experiment lasted for 16 weeks. In order to ensure that participants in the experimental (game-based) and control groups (nongame based or conventional) had the same knowledge, participants in both groups were asked to appear in a pretest. All the participants in both groups received teaching through conventional teaching methods until week 10 of the semester. However, the experimental and control groups were taught using 2 diverse teaching methods over the next 6 weeks (weeks 11‐16). During these 6 weeks, the participants in the experimental group experienced gamification to facilitate their learning of the topics taught during this period (see Figure 1 and Figure 2 to view the snapshots of one of the games used to teach cybersecurity), while the participants in the control group continued their learning of the same topics through conventional teaching methods.

**Figure 3.**
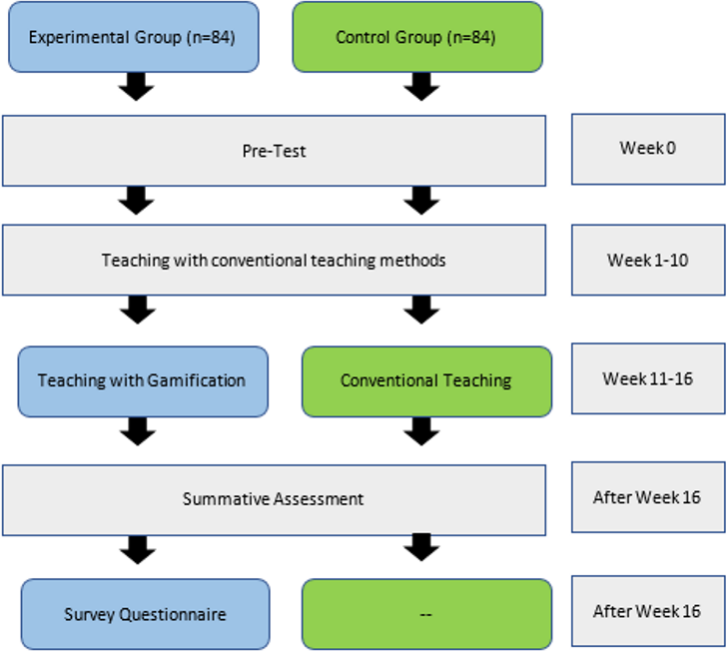
Experiment process.

After the 16-week teaching-learning process, both groups took an achievement test for the full term with some questions dedicated to assessing the topics covered in weeks 11-16 (the intervention time when the 2 groups of participants were taught using different teaching strategies, ie, game based and conventional). At the same time, a survey questionnaire was administered in the experimental group to assess their acceptability of gamification to support their learning in a digital literacy course (see Figure 3 for the graphical flow of the experimental process of this study). The study was authorized by the ethics committee of the corresponding author’s university. All the participants provided written informed consent before their participation in the study, and they did not receive any monetary or other sort of compensation.

### Game Application and Elements

The experimental group was introduced to NOVA Labs, a digital platform that engages learners in games and interactives that foster authentic scientific exploration. This platform incorporated several gamification elements to enhance students’ engagement and motivation:

1. Points system: Each task or activity within the course was assigned a point value. Students accumulated points for every completed activity, which contributed to their overall score and ranking on the leaderboard.
2. Badges: Students could earn badges for completing various tasks and reaching milestones. For instance, badges were awarded for completing weekly assignments on time, achieving high scores on quizzes, and participating in group discussions and collaborative projects.
3. Leaderboards: A dynamic leaderboard was displayed within the application, showcasing the top performers in the class. This element fostered a sense of competition and encouraged students to improve their performance to climb the ranks.
4. Challenges and quests: The course content was structured into thematic challenges and quests. Each week, students embarked on a new quest, which consisted of a series of tasks and activities related to the week’s learning objectives. Completing a quest unlocked new content and additional rewards.

The application supporting these gamification elements was user-friendly and accessible through both desktop and mobile devices. It featured an intuitive interface that guided students through their learning journey. Key functionalities included:

1. Dashboard: A personalized dashboard where students could track their progress, view earned badges, and see their current standing on the leaderboard.
2. Interactive lessons: Multimedia-rich lessons incorporating videos, interactive simulations, and practice exercises.
3. Real-time feedback: Immediate feedback on quizzes and assignments to help students identify areas for improvement.
4. Collaboration tools: Features enabling group work and peer-to-peer interactions, such as discussion forums and collaborative project spaces.

### Data Collection Tools

The study mainly used quantitative data collection methods. The data were collected through three tools: (1) pretest, (2) posttest, and (3) achievement. The aim of the pretest of academic achievement was to check if participants in the experimental and control groups fulfilled the same minimum criteria of prior knowledge and skills in digital literacy. While the purpose of the posttest was to assess participants’ assessment of digital literacy taught throughout the semester and to investigate whether there were differences between the 2 groups (experimental and control) using different teaching approaches, that is, game based and conventional, during weeks 11‐16 of the semester. These tests were developed by the instructor of the course and were validated by 2 senior professors of digital literacy with more than 10 years’ experience of teaching courses in the area of digital literacy.

The survey questionnaire used in this study was administered to the participants in the experimental group only. It was aimed to measure participants’ perceptions of gamification regarding their perceived usefulness, ease of use, and their intension to use gamification in future. This was modified from a scale developed by Ghani et al [30]. It consisted of 20 items such as “The educational digital game will improve my learning performance,” “I find the educational digital game is easy to use,” and “Studying using the educational digital game is a good idea.” (see Multimedia Appendix 1). The responses for the items were scored on a 5-point scale, with 1, 2, 3, 4, and 5 representing “strongly disagree,” “disagree,” “neutral,” “agree,” and “strongly agree,” respectively. The internal consistency of the scale was found to be acceptable with a Cronbach α value of 0.81 [31].

### Data Analysis

All data preprocessing and analyses were conducted using SPSS (version 21; IBM Corp). Statistical assumptions for parametric tests were checked and confirmed before running the main analyses [31]. Data analysis included descriptive statistics and inferential analysis including independent sample *t* test, 1-way between-subject ANOVA, and linear regression. Prior to conducting inferential statistical analyses, we tested for the assumptions underlying each statistical test. For the independent sample *t* test and ANOVA, we assessed the normality of data distribution using the Shapiro-Wilk test and ensured homogeneity of variances through Levene test. Additionally, the assumptions of linearity, independence of residuals, and homoscedasticity were confirmed for regression analysis. Moreover, Cronbach α was assessed to check the reliability of the instruments used.

### Ethical Considerations

This randomized controlled trial study was approved by the standing committee for Scientific Research Ethics of King Saud University (approval KSU-HE-22‐871). However, the trial was not registered by a clinical trial registration organization as it did not involve an explicit medical treatment.

## Results

This study primarily aimed to investigate the impact of gamification on students’ learning outcomes in a digital literacy course and to understand their perceptions and acceptance of gamification for their learning. The main results of this study are presented question-wise in the following sections.

### Impact of Gamification on Learning Outcomes

Our first question was focused on investigating whether there was any significant impact of the gamified learning experience on students’ learning outcomes. In specific terms, we wanted to check if there were significant differences in the academic achievement between the students who were taught using the gamified learning approach and those who were taught using conventional teaching methods in a digital literacy course at the undergraduate level. This question was analyzed using two main variables: (1) students’ scores in the achievement test conducted at the end of the term as dependent variable in the analysis and (2) students’ group (control and experimental) as an independent variable in the analysis. The dependent variable was measured on a scale with quantitative values ranging from 0 to 25, while the independent variable was recorded as a nominal variable with only 2 possible values: 1 representing the control group and 2 representing the experimental group.

The results of an independent-sample *t* test indicated that there was significant differences in students’ academic performance between those who were taught using conventional teaching methods (median 15.87, SD 2.15) and those whose learning was supported with gamification (mean 21.00, SD 1.88) in a digital literacy class *(t*_166_=−16.435; *P*=.001; Table 1). In simpler words, the experimental group performed better than the control group in the academic achievement test that was conducted as a summative assessment in the class. Overall, this finding suggests that the use of the gamification approach in the teaching of digital literacy proves to be an effective instructional approach at the undergraduate level.

**Table 1.** Independent *t* test results to test the impact of gamification.

		Participants, n (%)	Mean (SD)	*t* test (*df*)	*P* value
**Group**				–16.435 (166)	.001
	Control	84 (50)	15.87 (2.15)		
	Experimental	84 (50)	21.00 (1.88)		

### Acceptance of Gamification by the Students

The second research question proposed in this study seeks to find the factors that influence the acceptance of students toward gamified learning experiences in a digital literacy course. Students’ acceptance of a gamified learning experience was measured in terms of their attitude toward gamification for learning digital literacy. This variable was measured through Likert scale (ordinal type variables) items and was computed by taking the average of all items within the scale. Overall, the results indicated that the participants reported a high level of satisfaction (mean 3.857, SD 0.61) with their gamified learning experience in a digital literacy course. Regarding the factors influencing the acceptance of gamification by the students, we tested factors including age and the major of their degrees. Depending on the nature of variables, that is, scale variables and nominal variables with more than 2 levels, we used different statistical tests for the analysis of this research question.

The results of simple linear regression and 1-way between-subject ANOVA revealed that the major of the degree program was not a significant factor for participants’ acceptance of gamification in a digital literacy class (*P*=.06). Similarly, a simple linear regression analysis did not find age as a significant predictor of acceptance of gamification (*P*=.06). All in all, these results suggest that neither age nor major of degree are factors for participants’ high level of acceptance of gamified learning. The participants in this study showed a positive attitude toward the acceptance of gamification in a digital literacy course regardless of their age or the academic discipline of their degrees.

### Effect of Learners’ Prior Experience With Gamification

In the third question of the study, we were interested to confirm if participants’ prior experience with gamification affects their reported acceptance of gamified learning for a digital literacy course. For the analysis of this question, participants’ acceptance score served as the dependent variable, which was measured as a scale type variable, while their prior experience served as the independent variable, which was measured as a nominal variable with three levels: “no experience,” “little experience,” and “extended experience.” About one-third of the participants (n=26, 31%) had no prior experience of gamification. A total of 33 (39.3%) the participants reported that they had little experience of using games for learning. Likewise, 29.8% (n=25) of the participants had extended prior experience of gamification.

Since the levels of the independent variable were more than two, a 1-way between-subject ANOVA was chosen for the analysis of this question. The ANOVA results showed that there was no statistically significant difference in participants’ acceptance of gamified learning for the digital literacy course in respect to their prior experience of gamification (*F*_2,81_=1.319; *P*=.27; Table 2 for details). Since the main result of the ANOVA analysis was found to be nonsignificant, further post hoc analysis was not needed. These results suggested that regardless of the participants’ differences in their prior experience of gamification (some were not exposed to this teaching strategy, while some were experienced with it), their current level of acceptance for the gamified learning experience of digital literacy was almost similar.

**Table 2.** ANOVA results showing the influence of prior experience of gamification.

		Sum of squares (*df*)	Mean square	*F* test (*df*)	*P* value
**Analyses**				1.319 (2,81)	.27
	Between groups	0.974 (2)	0.487		
	Within groups	29.914 (81)	0.369		
Total		30.888 (83)	—^a^	—	—

a Not applicable.

## Discussion

### Principal Results

The ultimate goal of the study was to investigate the impact and acceptance of gamification in a digital literacy course at the undergraduate level. To achieve this goal, we addressed 3 research questions. The findings of these questions are discussed below.

For RQ1, we found that there was a significant difference in academic performance between students who were taught using conventional teaching methods and those who experienced gamified learning. Specifically, the experimental group, which was exposed to gamification, achieved a higher mean score (21.00) compared to the control group (mean 15.87), which received traditional teaching methods. This finding supports the notion that gamification can be an effective educational tool to enhance learning outcomes [32]. This finding has practical implications for educators and instructional designers. By implementing the game-based elements into the learning environment, instructors can potentially construct a more engaging and satisfying learning environment for learners [33]. Gamification techniques, such as scoring, badges, leaderboards, and interactive challenges, may foster a sense of competition, achievement, and enjoyment, helping to achieve better learning outcomes.

The data for this study show a high level of satisfaction (mean 3.857) with the gamified learning experience in a digital literacy course. The mean value being close to the upper end of the Likert scale (5) suggests that students generally had a positive attitude toward gamification as a learning approach. Our results are not different to the results of other studies that provide evidence for learners’ higher level of satisfaction with gamified learning [34-38]. This positive attitude is a promising indicator of the potential effectiveness of gamification in enhancing student engagement and motivation.

The study findings demonstrate that age is not a significant factor influencing students’ acceptance of gamified learning experiences. In this context, it means that students across different age groups showed similar levels of acceptance toward gamification. Although this finding suggests that gamified learning experiences can be suitable for a diverse range of age groups within the undergraduate level, it is important to acknowledge that the limited age range (18-22 years) of the participants in this study restricts our ability to draw strong conclusions about the impact of age on gamification acceptance. In addition, the finding of this study about age is different from what is suggested by Denden et al [38]. They found that “age” moderates the relationship between their experience of participating in a gamification program and perceived self-efficacy, such that it exerts a greater influence on older people.

Further, we found that the major of degree programs does not significantly impact students’ acceptance of gamification in a digital literacy course. In other words, regardless of their academic disciplines, students were similarly receptive to the gamified learning approach. This result is encouraging, as it suggests that gamification can be applied in various subject areas without compromising its acceptance among students.

The nonsignificant effects of age and the major of degree on students’ acceptance of gamified learning experiences have practical implications. It indicates that gamification has broad applicability and can be effectively integrated into digital literacy courses, irrespective of students’ demographic characteristics or academic backgrounds. Educators can use gamification as a versatile tool to enhance student engagement and motivation across diverse student populations.

Lastly, we found that the participants had varying levels of prior experience with gamification. However, there was no statistically significant difference in participants’ acceptance of gamified learning based on their prior experience with gamification. The 1-way between-subject ANOVA did not show any significant effect of prior experience (with 3 levels: no experience, little experience, and extended experience) on participants’ acceptance scores. In other words, whether participants had no exposure to gamification or considerable experience with it, their acceptance of gamified learning in a digital literacy course remained similar. It suggests that even students who have never been exposed to gamified learning strategies can still embrace and appreciate the approach. Additionally, students with previous experience with gamification did not necessarily have a more positive attitude toward it compared to their peers who had no experience. This implies that the effectiveness and acceptance of gamified learning can extend to a wide range of students with different levels of exposure to gamification.

### Limitations

While this study contributes valuable insights into the impact and acceptance of gamification in a digital literacy course at the undergraduate level, it is essential to acknowledge certain limitations. First, the research was conducted in a specific educational context, which may restrict the generalizability of the findings to other disciplines or educational levels. The use of convenience sampling, while practical for this study, further constrains the diversity of the participant pool. This sampling technique can lead to selection bias, as the sample may not be representative of the larger population. Future studies should aim to replicate this research with more diverse populations to explore the potential differences in gamification acceptance across various demographic and cultural groups.

The participant group, consisting of 168 native Arab speakers aged between 18 and 22 years from a single university, represents a fairly homogenous demographic. This homogeneity, while facilitating a focused analysis within this specific context, limits the broader applicability of our results. Another critical limitation arising from this homogeneity is the reduced variability in cultural background and academic exposure. Given the relatively homogeneous nature of our sample, caution should be exercised in extrapolating our conclusions to populations with greater age diversity or differing backgrounds. Future research endeavors should strive to recruit participants from a wider age spectrum to better understand the influence of age on acceptance of gamification in educational contexts. Additionally, exploring cultural and contextual factors beyond age could provide further insights into the acceptance and effectiveness of gamified learning experiences across diverse learner populations.

In addition, the study focused on a single course, and the participants were from a particular institution, which may limit the diversity of the sample. Additionally, the self-reported nature of some data, such as prior experience and acceptance scores, may have introduced response bias. Future research could address these limitations by conducting similar investigations in diverse educational settings with larger and more representative samples. Future studies could also explore the long-term effects of gamified learning experiences on students’ retention of knowledge, skill development, and continued motivation in subsequent courses or academic years. Longitudinal research can offer deeper insights into the sustained benefits of gamification in educational settings.

### Conclusions

In conclusion, this study sheds light on the impact and acceptance of gamification in a digital literacy course at the undergraduate level. The findings of our study have both theoretical and practical implications. The research reveals that gamified learning experiences positively influence students’ academic performance, leading to higher achievement compared to conventional teaching methods. This study also demonstrates that the students’ level of acceptability for gamified learning is not affected by prior experience of gamification. Educators and instructional designers can leverage the insights gained from this study to create more engaging and effective learning environments that foster student motivation and satisfaction. As the educational landscape continues to evolve, the integration of gamification into pedagogical practices stands as a promising approach to enriching the learning experiences of students in digital literacy courses and beyond.

## Supplementary material

10.2196/52017 Multimedia Appendix 1 Survey questionnaire.

## Abbreviations

SDT: self-determination theory
TAM: telephone acceptance model
