# Supplementary material for: The Impact and Acceptance of Gamification by Learners in a Digital Literacy Course at the Undergraduate Level: Randomized Controlled Trial
Source: JMIR Serious Games. 2024 Aug 23;12:e52017. doi: 10.2196/52017 (PMC11363743; doi:10.2196/52017)
Supplement: Multimedia Appendix 1 [file games-v12-e52017-s001.docx]

## Appendix A. Survey Questionnaire

**Q1.** What is your gender?

🞎 Male

🞎 Female

**Q2.** How old are you?

_____ years.

**Q3.** What is your major (program of study)? ___________

**Q4.** Please indicate your prior experience (before participating in the current study) of using gamification for learning in **any** subject/content area?

🞎 I have never used gamification before.

🞎 I have used gamification a couple of times.

🞎 I use gamification on a regular basis.

**Q5.** Considering your recent experience with the gamification in digital literacy course, please indicate your level of agreement (1=Strongly disagree, 2=Disagree, 3=Neutral, 4=Agree, and 5=Strongly agree) with the following statements:

|  | **Strongly Disagree** | **Disagree** | **Neutral** | **Agree** | **Strongly Agree** |
| --- | --- | --- | --- | --- | --- |
| The educational digital game will improve my learning performance. | 🞎 | 🞎 | 🞎 | 🞎 | 🞎 |
| The educational digital game will increase academic productivity. | 🞎 | 🞎 | 🞎 | 🞎 | 🞎 |
| The educational digital game could make it easier to study course content. | 🞎 | 🞎 | 🞎 | 🞎 | 🞎 |
| The educational digital game will enhance the effectiveness of learning. | 🞎 | 🞎 | 🞎 | 🞎 | 🞎 |
| I find the educational digital game useful. | 🞎 | 🞎 | 🞎 | 🞎 | 🞎 |
| I find the educational digital game is easy to use. | 🞎 | 🞎 | 🞎 | 🞎 | 🞎 |
| Learning how to use the educational digital game is easy for me. | 🞎 | 🞎 | 🞎 | 🞎 | 🞎 |
| It is easy to become skillful in using the educational digital game. | 🞎 | 🞎 | 🞎 | 🞎 | 🞎 |
| My interaction with the educational digital game is clear. | 🞎 | 🞎 | 🞎 | 🞎 | 🞎 |
| My interaction with educational digital game is understandable. | 🞎 | 🞎 | 🞎 | 🞎 | 🞎 |
| It will be easy for me to find information through the educational digital game. | 🞎 | 🞎 | 🞎 | 🞎 | 🞎 |
| Studying using the educational digital game is a good idea. | 🞎 | 🞎 | 🞎 | 🞎 | 🞎 |
| I feel positive towards the use of the educational digital game. | 🞎 | 🞎 | 🞎 | 🞎 | 🞎 |
| I believe that the educational digital game helps to be more engaged in learning. | 🞎 | 🞎 | 🞎 | 🞎 | 🞎 |
| I generally favour the use of the digital game for learning. | 🞎 | 🞎 | 🞎 | 🞎 | 🞎 |
| I believe that it is a good idea for me to use this educational digital game for my future coursework. | 🞎 | 🞎 | 🞎 | 🞎 | 🞎 |
| I intend to frequently use the digital game to learn digital literacy. | 🞎 | 🞎 | 🞎 | 🞎 | 🞎 |
| I intend to use the educational digital game heavily. | 🞎 | 🞎 | 🞎 | 🞎 | 🞎 |
| I intend to use educational digital game throughout this semester and the next. | 🞎 | 🞎 | 🞎 | 🞎 | 🞎 |
| I intent repetitively use the educational digital game as often as possible. | 🞎 | 🞎 | 🞎 | 🞎 | 🞎 |
